# Reproductive Toxicity Effects of Phthalates Based on the Hypothalamic–Pituitary–Gonadal Axis: A Priority Control List Construction from Theoretical Methods

**DOI:** 10.3390/ijms26157389

**Published:** 2025-07-31

**Authors:** Botian Xiao, Hao Yang, Yunxiang Li, Wenwen Wang, Yu Li

**Affiliations:** 1 College of Environmental Science and Engineering, North China Electric Power University, Beijing 102206, China; xbt13785387874@163.com (B.X.); yh13601614368@163.com (H.Y.); 120222232055@ncepu.edu.cn (Y.L.); liyuxx8@hotmail.com (Y.L.); 2 MOE Key Laboratory of Resources and Environmental System Optimization, North China Electric Power University, Beijing 102206, China

**Keywords:** phthalate esters, adverse outcome pathway, reproductive toxicity, priority control list, molecular dynamics simulation, machine learning

## Abstract

Phthalate esters (PAEs), frequently detected in various environmental media, are associated with multiple health issues, particularly reproductive toxicity. This study employed molecular docking and molecular dynamics simulations to investigate the reproductive toxicity risk of 22 PAEs on the regulation of the hypothalamic–pituitary–gonadal (HPG) axis. Analysis revealed that when the carbon number of PAEs was the same, those with branched side chains exhibited more pronounced reproductive toxicity risks. In PAE molecules with branched side chains, reproductive toxicity risk was inversely proportional to the number of carbon atoms. Furthermore, five PAE molecules with unacceptable risk (DIPRP, DMEP, DMP, DPP, and DUP) and four key indicators were proposed. Key descriptors influencing PAEs’ reproductive toxicity risks were identified as Infrared and ATSC8e by machine learning analysis. Furthermore, carbonyl structure, substituent position, and electronegativity of PAE molecules are critical factors influencing PAE-induced reproductive toxicity risks via the HPG axis. This study provides a theoretical basis for further investigation of PAE-induced reproductive toxicity risk on the HPG axis, which facilitates the development of risk mitigation strategies for PAEs’ reproductive toxicity and provides novel perspectives and approaches for exploring the molecular mechanisms underlying the endocrine effects of emerging contaminants such as PAEs.

## 1. Introduction

Phthalate esters (PAEs), derivatives of phthalic acid, are characterized by a distinctive odor, insolubility in water, and solubility in most organic solvents [1]. Based on the length of their ester alkyl chains, PAEs are broadly classified into two categories: high molecular weight PAEs (7–13 carbon atoms) and low molecular weight PAEs (3–6 carbon atoms) [2]. PAEs are commonly present in five typical plastics: polyamide, polypropylene, polyvinyl chloride, polyethylene terephthalate, and polyethylene [3]. The environmental presence of PAEs is closely associated with the extent of plastic pollution. In recent years, the continuous increase in global plastic production, combined with the aging and degradation of plastic products in environmental media, has resulted in the persistent leaching of additives such as PAEs from plastic waste [4]. Additionally, due to their volatility, PAEs are readily released from various products and enter multiple environmental media [5]. Recent studies have detected PAEs at relatively high concentrations in various environmental media worldwide, including air [6], indoor dust [7], road dust [8], water bodies [9], and sediments [10]. Numerous studies have demonstrated that certain PAEs can induce developmental toxicity, reproductive toxicity, neurotoxicity, and even carcinogenicity, teratogenicity, and mutagenicity [11]. This issue has attracted widespread global attention; therefore, the potential environmental effects and health impacts caused by PAE exposure still require priority focus to mitigate their ongoing threat to human health.

Due to the widespread presence of PAEs in various environmental media, they have been identified as typical endocrine-disrupting chemicals. PAEs can enter the human body through direct exposure routes, such as inhalation and dermal contact, or indirect exposure via dietary intake, leading to a range of adverse effects on the reproductive and developmental systems [12,13]. Studies have demonstrated associations between PAE exposure and reproductive disorders in both males and females. In males, exposure to PAEs has been linked to multiple reproductive issues, including decreased sperm concentration and motility, disruption of endogenous hormone levels, altered sperm morphology, and DNA damage [14]. Wang et al. [15] reported that exposure to PAEs at environmental levels may impair semen quality in males. In females, PAE exposure can adversely affect reproductive health by disrupting the normal physiological functions of the ovaries and uterus, leading to health risks such as infertility, thyroid dysfunction, and endometriosis [16]. Mu et al. [17] demonstrated that exposure to PAEs during pregnancy significantly increases the risk of clinical miscarriage. Compared to adults, children are more susceptible to the effects of PAEs. Related studies have indicated that PAE exposure in children can result in endocrine disorders (e.g., precocious puberty, thyroid diseases), allergic diseases (e.g., allergic rhinitis, asthma), and neurological disorders (e.g., intellectual disabilities, autism) [18]. Hashemipour et al. [19] reported that the intensity of PAE exposure is associated with precocious puberty in girls. Furthermore, they found that PAEs can disrupt bone modeling and remodeling, resulting in detrimental effects on bone tissue [20]. Additionally, PAEs inhibit calcium signaling in human nicotinic acetylcholinesterase, thereby reducing neural transmission efficiency [21]. In summary, PAEs pose significant and unavoidable health hazards to humans, particularly impairing reproductive functions. Therefore, investigating the reproductive toxicity risks induced by PAE exposure is essential to mitigate their adverse effects on the normal human reproductive system.

Due to their structural characteristics and inherent chemical properties, PAEs have been shown to regulate organismal development by mimicking hormonal signals or binding to endocrine receptors. Upon entering the human body, PAEs interfere with multiple hormonal signaling pathways, such as the hypothalamic–pituitary–gonadal (HPG) axis, disrupting its normal function. However, studies investigating the interference of PAEs with the endocrine system via activation of peroxisome proliferator-activated receptors (PPARs) and aryl hydrocarbon receptor (AhR) remain limited [22]. The HPG axis is a critical regulator of the normal development and function of the human reproductive system [23]. As a key endocrine pathway controlling sex hormone secretion and reproductive functions, it serves as a pivotal axis and important target mediating reproductive toxicity risks induced by PAE exposure. The HPG axis is indispensable for regulating the reproductive systems of both sexes [22]. The HPG axis consists of three components: the hypothalamus, pituitary gland, and gonads. Gonadotropin-releasing hormone (GnRH), secreted by the hypothalamus, binds to GnRH receptors in the anterior pituitary via the hypophyseal portal circulation, stimulating the secretion of luteinizing hormone (LH) and follicle-stimulating hormone (FSH). After entering the bloodstream, LH and FSH act on different cells within the gonads to regulate reproductive functions [22]. PAEs can exert hormonal and other adverse effects on all three components of the HPG axis, including abnormal secretion of GnRH, estrogen (E2), gonadotropins, and androgens, leading to endocrine dysfunction [24]. Moreover, abnormalities at any site within the HPG axis may result in detrimental reproductive outcomes, such as testicular damage [25], ovarian insufficiency [26], or increased risk of miscarriage [27]. Therefore, constructing an adverse outcome pathway (AOP) for the HPG axis to elucidate the toxic pathways and mechanisms of PAE exposure is crucial for understanding their adverse effects on the HPG axis and comprehensively assessing their reproductive toxicity risks.

The HPG axis is the primary pathway mediating the reproductive toxicity risks of PAEs in humans [28]. However, studies exploring the toxic mechanisms of PAEs on the HPG axis remain limited, and systematic evaluations of the reproductive toxicity risks posed by various PAEs on this axis are scarce. Therefore, to comprehensively investigate the reproductive toxic risks and underlying mechanisms of different PAEs on the HPG axis, this study focuses on PAE molecules as the research subject. (1) Multiple methods, including molecular docking (MD), molecular dynamics simulation (MDs), integrated index methods, and machine learning, were employed to quantify the reproductive toxicity risks of PAEs on the HPG axis; (2) a hierarchical evaluation system for assessing the reproductive toxicity risks of PAEs on the HPG axis was established, followed by cross-sectional and longitudinal analyses to validate the system. Differences in reproductive toxicity risks associated with exposure to various PAEs were elucidated, and pathways exhibiting significant reproductive toxicity risks were identified; (3) a priority control list for the reproductive toxicity risks of PAEs on the HPG axis was developed using equal interval classification and machine learning methods, and its validity was subsequently confirmed. This study elucidated the toxic mechanisms of PAEs and established a corresponding priority control list, which holds significant importance for comprehensively assessing the reproductive toxicity risks of various PAEs on the human HPG axis and identifying key pathways through which PAEs affect this axis. The findings contribute to promoting and accelerating the development of strategies to mitigate the reproductive toxicity risks of PAEs and provide theoretical support for the comprehensive evaluation of reproductive toxicity risks on the HPG axis under exposure to multiple PAEs.

## 2. Results and Discussion

### 2.1. Calculation of Graded Indicators for the Evaluation System of Reproductive Toxicity Risks on the HPG Axis Under PAE Exposure

Calculation of the third-level evaluation index for the reproductive toxicity risk assessment system of the HPG axis under PAE exposure was conducted. In this study, the method described in Section 3.3 was employed to calculate the binding free energy values between receptor proteins and 22 PAE molecules at the initiating and key events of pathways ①, ②, ③, ④, ⑤, ⑥, ⑦, and ⑧, with absolute values taken. Using the method outlined in Section 3.4.1, the weights of initiating and key events were calculated as 0.5:0.5, and the integrated effect values of each HPG axis pathway under PAE exposure were determined. These integrated effect values serve as the third-level evaluation index for the reproductive toxicity risks of PAEs on the HPG axis (Table S1).

Calculation of the second-level evaluation index in the reproductive toxicity risk assessment system of the HPG axis under PAE exposure was performed. Using the method described in Section 3.4.2, the third-level evaluation index values underwent inverse interval transformation. Subsequently, the CRITIC objective weighting method and integrated index method were applied to comprehensively calculate the second-level evaluation index for eight pathways: hypothalamic pathways (①, ②, ③, ④), pituitary pathways (⑤, ⑥), and gonadal pathways (⑦, ⑧). The second-level evaluation index A-B1 (hypothalamus) was derived from the integrated third-level evaluation indices A-B1-C1, A-B1-C2, A-B1-C3, and A-B1-C4, corresponding to reproductive toxicity pathways ①, ②, ③, and ④ of the HPG axis under PAE exposure, respectively. The respective weights of these third-level evaluation indices are 20.90%, 27.30%, 30.90%, and 20.90%. Similarly, the second-level evaluation index A-B2 (pituitary) was derived from the integrated third-level evaluation indices A-B2-C5 and A-B2-C6, corresponding to reproductive toxicity pathways ⑤ and ⑥ of the HPG axis under PAE exposure, respectively. The respective weights of these third-level evaluation indices are 47.01% and 52.99%. The second-level evaluation index A-B3 (gonads) was derived from the integrated third-level evaluation indices A-B3-C7 and A-B3-C8, corresponding to reproductive toxicity pathways ⑦ and ⑧ of the HPG axis under PAE exposure, with respective weights of 55.90 and 44.10%. The second-level evaluation index values for the reproductive toxicity risks of PAEs on the HPG axis were calculated using the integrated index method (Table S1).

Calculation of the first-level evaluation index in the reproductive toxicity risk assessment system of the HPG axis under PAE exposure was conducted. Based on the second-level evaluation indices of reproductive toxicity risks on the HPG axis under PAE exposure, the method described in Section 3.4.3 was used to calculate the first-level evaluation index using the CRITIC objective weighting method and the integrated index method. The first-level evaluation index A was derived from the integrated second-level evaluation indices A-B1, A-B2, and A-B3, which represent the comprehensive reproductive toxicity risk values of the hypothalamus, pituitary, and gonads, respectively, under PAE exposure. The corresponding weights of these indicators are 30.36, 30.21, and 39.42%, respectively. The first-level evaluation index value for the reproductive toxicity risks of PAEs on the HPG axis was calculated using the integrated index method (Table S1).

### 2.2. Longitudinal Analysis of Graded Indicators in the Evaluation System of Reproductive Toxicity Risks on the Hpg Axis Under PAE Exposure

#### 2.2.1. Longitudinal Analysis of the Evaluation System for Reproductive Toxicity Risks on the HPG Axis Under PAEs Exposure

**Figure 1:**
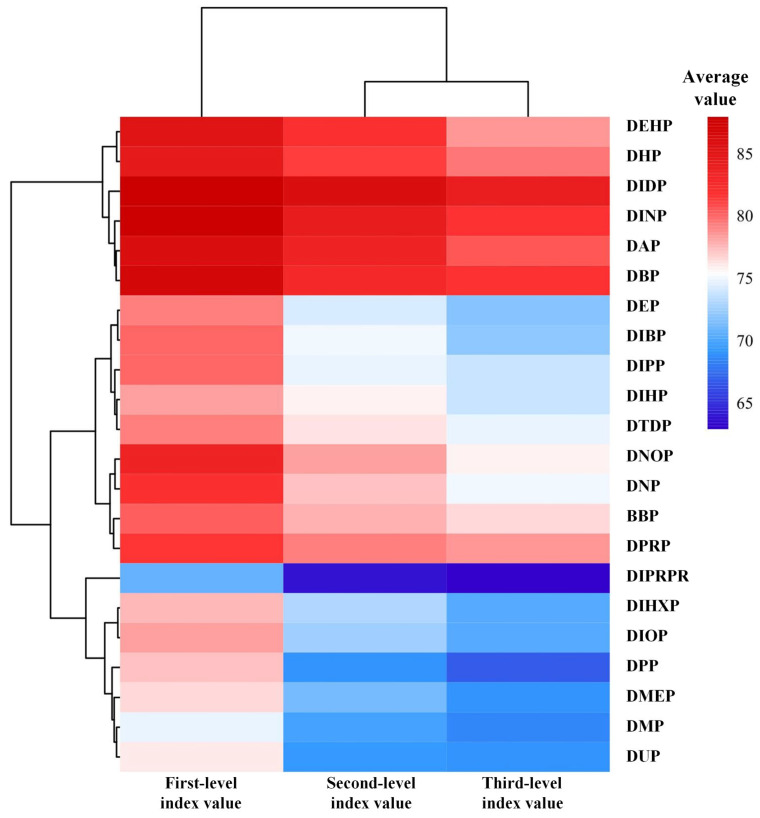
Analysis of average values of reproductive toxic risk evaluation indicators at various levels of the HPG axis under PAE exposure.

To investigate the differences in reproductive toxicity risks on the HPG axis under exposure to different PAE molecules, a longitudinal feature analysis was conducted using the evaluation system of reproductive toxicity risks on the HPG axis under PAE exposure. Based on the graded indicator calculation table in this evaluation system (Table S1), the average values of evaluation indicators at each level were calculated for each PAE molecule to characterize the reproductive toxicity risk values at different levels of the HPG axis. As described in Section 3.3, smaller evaluation indicator values correspond to stronger reproductive toxicity risks of the respective PAE molecules on the HPG axis. Subsequently, ranking analysis was performed (Figure 1). The results showed that the average values of reproductive toxicity risk evaluation indicators at various levels of the HPG axis under exposure to 22 PAE molecules ranged from 88.033 to 63.003, indicating significant differences in reproductive toxicity risks on the HPG axis among different PAE molecules.

Figure 1 revealed that the rankings of reproductive toxicity risks at various levels of the HPG axis for each PAE molecule were similar. The Friedman test was employed to perform non-parametric analysis and post hoc multiple comparisons on the rankings of reproductive toxicity risks across different levels for PAE molecules. The results showed no significant differences in the average ranking values of reproductive toxicity risk evaluation indicators at various levels (all *p*-values = 1.000, well above the significance threshold of 0.05). This indicates that, statistically, no significant differences were observed in the rankings of reproductive toxicity risk evaluation indicators at different levels for the same PAE molecule, demonstrating consistency in the average rankings of reproductive toxicity risk evaluation indicators across the 22 PAE molecules. Furthermore, the ranking results of reproductive toxicity risk evaluation indicators for the 22 PAE molecules (Figure 1) suggest that the strength of PAEs’ reproductive toxicity risks may be related to the ester side chain structure and the carbon number of the molecules. Taking DIPRP and DPRP as examples, analysis revealed that DIPRP possesses a branched ester side chain, whereas DPRP has a linear ester side chain; despite having the same carbon number, DIPRP exhibited stronger toxicity risks than DPRP. Therefore, it is preliminarily hypothesized that, among PAE molecules with the same carbon number, those with branched structures exhibit more pronounced reproductive toxicity risks. Furthermore, among PAE molecules with branched side chains, the reproductive toxicity risk of DIPRP, which contains five carbons, is significantly higher than that of DIDP, which contains ten carbons. Therefore, it can be preliminarily inferred that, for PAE molecules with branched side chains, those with a greater number of carbons exhibit weaker reproductive toxicity risks. However, for PAE molecules with linear side chains, no significant correlation was observed between carbon number and the strength of reproductive toxicity risks.

#### 2.2.2. Longitudinal Validation of the Evaluation System for Reproductive Toxicity Risks on the HPG Axis Under PAE Exposure

**Figure 2:**
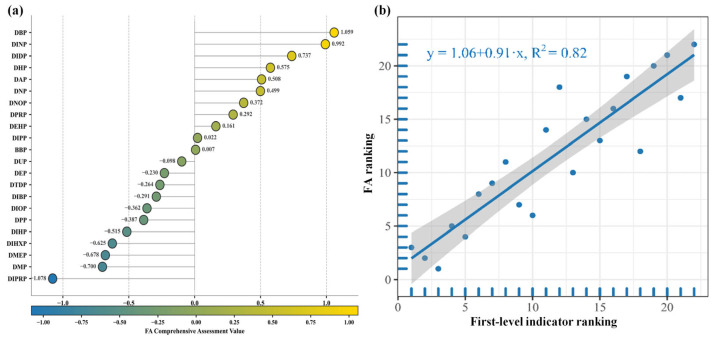
Linear regression plot of FA comprehensive scores (**a**) and first-level evaluation index rankings (**b**) of reproductive toxic risks on the HPG axis under PAE molecules exposure.

To further elucidate the comprehensive characteristics of reproductive toxicity risks on the HPG axis induced by 22 PAE molecules, factor analysis (FA) comprehensive scores were calculated based on the second-level evaluation indices, and the procedure is described in Section 3.5 (Table S2). As the graded evaluation indicators of reproductive toxicity risks on the HPG axis under PAE exposure are all negative indicators, higher FA comprehensive scores indicate weaker toxic risks of PAEs on the HPG axis. Therefore, as shown in Figure 2a, the top six PAE molecules with the highest FA comprehensive scores exhibited the least significant reproductive toxic risks on the HPG axis, ranked as DBP, DINP, DIDP, DHP, DAP, and DNP, respectively. Conversely, the six PAE molecules with the lowest FA comprehensive scores demonstrated the most significant reproductive toxic risks, ranked as DIPRP, DMP, DMEP, DIHP, and DPP. These results are highly consistent with the average ranking outcomes of reproductive toxicity risk evaluation indicators at various levels of PAE molecules presented in Section 2.2.1 of this study. Correlation analysis was conducted between the FA comprehensive score rankings and the first-level evaluation index rankings of PAE molecules on the HPG axis, as shown in Figure 2b. The analysis revealed a coefficient of determination R^2^ of 0.823 (>0.75), indicating strong consistency between the two rankings. This finding validates the rationality of the reproductive toxicity risk evaluation system based on the HPG axis under PAE exposure constructed in this study.

To further validate the relationship between the strength of reproductive toxicity risks on the HPG axis under PAE molecules exposure and the ester side chain structure and carbon number, molecular structure analyses were conducted. Firstly, analysis of DIHP and DHP revealed that both molecules contain seven carbons; DIHP possesses a branched side chain, whereas DHP has a linear side chain, with DIHP exhibiting higher reproductive toxicity risks than DHP. Similarly, analysis of DIBP and DBP showed that both contain four carbons; DIBP has a branched side chain, while DBP has a linear side chain, and DIBP also exhibits higher reproductive toxicity risk than DBP. Li et al. [29] also confirmed that the branched side chain structure of PAE molecules increases their reproductive toxicity risks. When the carbon number is the same, PAE molecules with branched side chains exhibit more pronounced toxic risks, which is consistent with the results of this study. Secondly, the side chains of DIDP, DINP, DEHP, DIHP, and DIBP are all branched structures, with carbon numbers of 10, 9, 8, 7, and 4, respectively. Their reproductive toxicity risks under exposure increase correspondingly, further validating the conclusion of this study that, among PAE molecules with branched side chains, reproductive toxicity risk is inversely proportional to carbon number. Finally, analysis of PAE molecules with linear side chains shows that the most toxic ones—DPP, DMP, and DMEP (ranked from highest to lowest reproductive toxicity risk)—have carbon numbers of five, one, and six, respectively. Conversely, the least toxic PAE molecules—DPRP, DHP, and DBP (ranked from highest to lowest toxicity risk)—have carbon numbers of three, seven, and four, respectively. Li et al. [29] also found that among PAEs molecules with linear side chains, those with carbon numbers of 5–6 exhibit the most significant reproductive toxicity risks; those with carbon numbers of 4 or 7 show moderate toxicity risk; and those with a carbon number of 3 exhibit the weakest toxicity risk. These findings are highly consistent with the conclusions of this study, further validating the accuracy and reliability of the PAE molecules identified here as having the highest reproductive toxicity risk. Furthermore, relevant studies have shown that molecular lipophilicity is significantly related to side chain structure [30]. Therefore, this study uses logKow values to characterize the lipophilicity of PAE molecules. Based on ChemDraw 2019 software, the logKow values of four PAE molecules with the strongest and weakest reproductive toxicity risks were calculated (Table S3). The results indicate that the logKow values of DIPRP, DPP, DMP, and DEMP are remarkably lower than those of DIDP, DINP, DBP, and DAP. This difference is notable considering that the initial events of the pathway constructed in this study involve PAE molecules interacting with receptor proteins on the cell membrane. It is hypothesized that PAE molecules with stronger reproductive toxicity risks have lower logKow values. This may be because PAE molecules with lower logKow values are less likely to penetrate the cell membrane, leading to greater accumulation on the membrane surface and thereby more significantly affecting the interaction between PAE molecules and membrane receptor proteins.

### 2.3. Cross-Sectional Analysis of Grading Indicators in the HPG Axis Reproductive Toxicity Assessment System Under PAE Exposure

To elucidate the differences in reproductive toxicity pathways of 22 PAEs and their risks on different human tissues, a cross-sectional statistical analysis was conducted based on the third- and second-level evaluation indices of the HPG axis reproductive toxicity risk grading assessment system under PAE exposure. Analysis of the average values of the HPG axis reproductive toxicity risk assessment indicators for these 22 PAEs (Table S4) revealed that, within the second-level evaluation index A-B1 of the HPG axis reproductive toxicity risk assessment system, the reproductive toxicity risks of A-B1-C4 and A-B1-C3 were significant and non-remarkable, respectively, under exposure to these PAEs. Within the second-level evaluation index A-B2, the reproductive toxicity risks of A-B2-C6 and A-B2-C5 were significant and non-significant, respectively. Within the second-level evaluation index A-B3, the reproductive toxicity risks of A-B3-C8 and A-B3-C7 were significant and non-significant, respectively. Furthermore, within the second-level evaluation indices, the reproductive toxicity risks of A-B3 and A-B1 were significant and non-significant, respectively. Based on these results, preliminary differences in reproductive toxicity risks among various evaluation indicators of PAEs were identified. However, to ensure that these findings were not due to chance, a further cross-sectional analysis of the grading indicators of HPG axis reproductive toxicity risks under PAE exposure was conducted.

#### 2.3.1. Analysis and Validation of the Third-Level Evaluation Index in the HPG Axis Reproductive Toxicity Risk System Under PAE Exposure

The method described in Section 3.6 was used to assign weights to 22 PAEs within the HPG axis reproductive toxicity risk evaluation indicators A-B1, A-B2, and A-B3. Based on the weighted results, the comprehensive Rank Sum Ratio (RSR) scores of the third-level evaluation indices of HPG axis reproductive toxicity risks under PAE exposure were calculated using the method outlined in Section 3.6 (Table S5). The RSR comprehensive scores and grading levels ranged from zero to one and I to III, respectively. A higher RSR score approaching one or a higher grading level indicates a more pronounced reproductive toxicity risk on the HPG axis under PAE exposure. As shown in Table S5, the RSR comprehensive score of the reproductive toxicity risk evaluation indicator A-B1-C4 (1.076) for PAEs was higher than those of A-B1-C1, A-B1-C2, and A-B1-C3. Similarly, the RSR score of A-B2-C6 (0.977) exceeded that of A-B2-C5, and the RSR score of A-B3-C8 (1.000) was higher than that of A-B3-C7. Accordingly, PAEs exhibited stronger reproductive toxicity risks on A-B1-C4, A-B2-C6, and A-B3-C8, consistent with the analysis results presented in Table S4. Additionally, the RSR comprehensive scores of reproductive toxicity risk evaluation indicators A-B1-C3 (0.349), A-B2-C5 (0.523), and A-B3-C7 (0.500) were the lowest; however, their grading levels were all classified as Level II, similar to other indicators. As shown in Table S4, except for the indicators A-B1-C4, A-B2-C6, and A-B3-C8, the third-level evaluation indices within A-B1, A-B2, and A-B3 of the HPG axis reproductive toxicity risks under PAE exposure were all non-significant. Therefore, future assessments of PAE-induced reproductive toxicity risks on the HPG axis should emphasize the binding interactions of PAE molecules with insulin-like growth factor 1 (IGF-1) and the IGF1R/IRS-1 complex, GNRH and the GNRH/G(s) complex, as well as INS and insulin receptors (INSR), to effectively evaluate the reproductive toxicity risk under PAE exposure.

#### 2.3.2. Analysis and Validation of the Second-Level Evaluation Index in the HPG Axis Reproductive Toxicity Risk System Under PAE Exposure

The method described in Section 3.6 was used to assign weights to 22 PAEs within the second-level evaluation indices A-B1, A-B2, and A-B3 of the HPG axis reproductive toxicity risks. Based on the weighting results, the RSR method was used to calculate the comprehensive RSR scores of the second-level evaluation indices under PAE exposure (Table S5). As shown in Table S5, the comprehensive RSR scores of the second-level evaluation indices ranged from 0.350 to 0.988, indicating significant differences in the risks of PAE exposure on the three second-level evaluation indices. Among them, the RSR score of A-B3 (0.988) under PAE exposure was higher than those of A-B1 and A-B2, suggesting that PAEs exert a stronger reproductive toxicity risk on the A-B3 indicator. This indicates that the gonads are the key target sites of PAE-induced reproductive toxicity risks on the HPG axis, consistent with the analysis results presented in Table S4. Additionally, under PAE exposure, the reproductive toxicity risk evaluation indicator A-B1 had the lowest comprehensive RSR score (0.350), and both A-B1 and A-B2 were classified as Level II. The combined RSR scores of A-B1 and A-B2 showed a considerable gap compared to that of A-B3. Therefore, the reproductive toxicity risks of PAEs on the A-B1 and A-B2 indicators are not significant. In other words, compared to the gonads, PAE exposure results in relatively minor reproductive toxicity risks on the hypothalamus and pituitary. Overall, the toxic risks of PAE molecules on human gonads warrant focused attention.

### 2.4. Priority Control List of HPG Axis Reproductive Toxicity Risks and Focused Attention List of Toxicity Indicators Under PAE Exposure

#### 2.4.1. Priority Control List of PAEs Reproductive Toxicity Risks Based on the Equal Interval Classification Method

To integrate the risk levels of HPG axis reproductive toxicity under exposure to 22 PAEs, this study proposes a priority control evaluation list for PAE-induced reproductive toxicity risks on the HPG axis. First, based on the first-level evaluation index values of HPG axis reproductive toxicity risks under PAE exposure, a comprehensive assessment was conducted using the equal interval classification method. The six equal-interval score ranges for the first-level evaluation index are as follows: [63.002, 66.544), (66.544, 70.086), (70.086, 73.627), (73.627, 77.169), (77.169, 80.710), and (80.710, 84.252], corresponding to scores of 6, 5, 4, 3, 2, and 1, respectively, where a higher score indicates a stronger reproductive toxicity risk under PAE exposure. PAEs within six intervals were assigned scores of 6, 5, 4, 3, 2, and 1, respectively, where higher scores indicated stronger reproductive toxicity risks under PAE exposure. Accordingly, based on the scoring results, PAEs with scores of 6 and 5 were classified as unacceptable risk, exhibiting strong reproductive toxicity risks on the HPG axis; those with scores of 4 and 3 were classified as potential risk, showing moderate reproductive toxicity risks on the HPG axis; and those with scores of 2 and 1 were classified as acceptable risk, exhibiting weaker reproductive toxicity risks on the HPG axis, although this does not imply the absence of reproductive toxicity risk (Table 1). Comparative analysis reveals significant consistency between the PAEs’ molecular risk classification results and the comprehensive reproductive toxicity risk scores presented in Section 2.2.2. Considering both evaluation results, this study highlights that the comprehensive reproductive toxicity risks on the HPG axis under exposure to DIPRP, DMP, DMEP, and DPP warrant focused attention. Sedha et al. [31] reported that among higher molecular weight PAEs, such as DINP, the adverse effects on the human reproductive system are relatively minor, whereas lower molecular weight PAEs like DMP exhibit greater reproductive toxicity risks. Furthermore, Heindel et al. [32] reported that DPP exhibits high toxic risks to both male and female reproductive systems, while DIPRP shows significantly higher toxicity to the female reproductive system compared to males. Both compounds can substantially reduce fertility, further validating the rationality of the conclusions drawn in this study. DMEP was confirmed as one of the most toxic PAEs as early as 1982 [33]. As a low molecular weight PAE, it exhibits significant reproductive toxicity risks in humans, affecting reproductive function and reducing fertility [34], which is consistent with the findings of this study.

Based on the priority control list of human reproductive toxicity risks under PAEs molecular exposure, five PAEs with comprehensive scores ranging from 5 to 6 were classified as posing unacceptable risks. It is recommended that their use be restricted or that molecular modification techniques be employed to reduce their reproductive toxicity risks without compromising their original functions. Additionally, ten PAEs with scores ranging from 3 to 4 were identified as having potential reproductive toxicity risks. It is recommended that the widespread use of these PAEs be controlled, and that the recycling and disposal of related plasticizer products be enhanced to prevent excessive environmental exposure and human intake through multiple pathways such as inhalation and dermal absorption. Although seven PAEs with scores of 1 to 2 were classified as having acceptable reproductive toxicity risks to humans, this does not imply the absence of reproductive health risks. Therefore, the recycling of plasticizer products containing these PAEs should also be emphasized, alongside extensive monitoring and detection efforts.

#### 2.4.2. Focused Attention List of PAEs Reproductive Toxicity Indicators Based on the Equal Interval Classification Method

To comprehensively assess the differences among various indicators of HPG axis reproductive toxicity risks induced by PAEs and to identify key indicators requiring focused evaluation under PAE exposure, this study employed the equal interval classification method to conduct a comprehensive assessment and scoring of the second- and third-level evaluation indices of HPG axis reproductive toxicity risks, based on the average values of each second- and third-level evaluation index. The six equal-interval score ranges for the second-level evaluation indices A-B1, A-B2, and A-B3 were as follows: [37.157, 49.206), (49.206, 61.254), (61.254, 73.303), (73.303, 85.352), (85.352, 97.400), and (97.400, 109.449]. For the third-level evaluation indices A-B1-C1, A-B1-C2, A-B1-C3, and A-B1-C4, the six equal-interval score ranges were [69.947, 83.925), (83.925, 97.903), (97.903, 111.882), (111.882, 125.860), (125.860, 139.838), and (139.838, 153.817]. The six equal-interval score ranges for the third-level evaluation indices A-B2-C5 and A-B2-C6 were as follows: [2.853, 24.982), (24.982, 47.111), (47.111, 69.241), (69.241, 91.370), (91.370, 113.499), and (113.499, 135.628]. For the third-level evaluation indices A-B3-C7 and A-B3-C8, the six equal-interval score ranges were [5.975, 23.426), (23.426, 40.878), (40.878, 58.329), (58.329, 75.780), (75.780, 93.232), and (93.232, 110.683]. Based on the scoring criteria outlined in Section 2.4.1, a list of key indicators requiring focused attention during the assessment of HPG axis reproductive toxicity risks under PAE exposure was constructed. As shown in Table 2, among the second-level evaluation indices, the reproductive toxicity risk of PAE exposure on A-B3 was the most significant; therefore, it was designated as a key indicator requiring focused attention. Secondly, A-B2 was classified as a general attention indicator, while the reproductive toxicity risk of PAEs exposure on A-B1 was the least significant and thus designated as a secondary attention indicator. Among the third-level evaluation indices, the reproductive toxicity risks of PAE exposure on A-B1-C4, A-B3-C8, and A-B1-C1 were the most significant and thus designated as key indicators for focused attention. The toxicity risks on A-B2-C5 and A-B3-C7 were the least significant and classified as secondary attention indicators, while the remaining indicators were considered general attention indicators. These results are consistent with the key impact indicators identified in Section 2.3, thereby validating the accuracy of the screening and evaluation results presented in this study.

Literature review indicates that E2 is secreted by ovarian cells of the gonads. PAEs exhibit estrogen-mimicking properties and can compete with endogenous E2 for binding to receptor proteins [35], resulting in hormonal imbalance and disruption of hormone signaling within the HPG axis. The above analysis indicates that PAEs primarily exert reproductive toxicity risks on humans by targeting the gonads. The gonads serve both as the toxic endpoint of PAE-induced reproductive toxicity risks via the HPG axis and as the starting point of negative feedback, further validating the accuracy of this study’s conclusion that A-B3 is a key indicator requiring focused attention. Moreover, existing literature indicates that PAEs can significantly alter IGF-1 concentration levels, leading to abnormal expression of the IGF-1 gene [36]. The specific binding of IGF-1 to the insulin-like growth factor 1 receptor/insulin receptor substrate 1 (IGF-1R/IRS-1) complex not only influences the synthesis and release of kisspeptin via the PI3K/Akt/mTOR signaling pathway [37], thereby maintaining reproductive system health and stability, but also regulates reproductive system function by inducing the release of PGE2 [38]. Shao et al. [39] demonstrated that overexpression of the IGF-1/PI3K/Akt/mTOR signaling pathway is a primary trigger for precocious puberty in mice. The above analysis further validates the reliability of this study’s conclusion that A-B1-C1 and A-B1-C4 are key indicators warranting focused attention. Finally, PAEs are closely associated with insulin (INS) synthesis and secretion disorders [40] as well as insulin resistance [41]. The specific binding of insulin to the INSR/IRS-1 complex activates the PI3K and MAPK signaling cascades and induces steroid biosynthesis [42]. Therefore, PAEs interfere with the insulin pathway to hinder steroid biosynthesis, exerting reproductive toxicity risks on humans, which fully supports the accuracy of designating A-B3-C8 as a key indicator requiring focused attention. In summary, during the comprehensive assessment of PAEs’ reproductive toxicity risks, key attention should be given to the indicators A-B3, A-B1-C4, A-B3-C8, and A-B1-C1. It is recommended that these indicators be considered as routine evaluation items. Conversely, secondary attention indicators could be reduced or omitted based on the actual research context of PAEs’ reproductive toxicity risks, thereby narrowing the scope of HPG axis reproductive toxicity risk assessment and enhancing the pertinence and accuracy of the study.

Considering both the priority control evaluation list for HPG axis reproductive toxicity risks under PAE exposure and the focused attention list of graded toxicity risk evaluation indicators established in this study, it was found that reproductive toxicity risks under exposure to DIPRP, DMP, DMEP, DPP, and DUP contributed most significantly, whereas those under exposure to DBP, DIDP, and DINP contributed relatively least. Moreover, the primary target site of PAE-induced reproductive toxicity risks in humans was identified as the gonads (A-B3). Significant risks were observed on the binding of insulin to its receptor (A-B3-C8) and on the interaction between insulin-like growth factor and the insulin-like growth factor receptor–insulin receptor substrate complex (A-B1-C1, A-B1-C4). These findings indicate that the key mechanism by which PAEs exert reproductive toxicity risks via the HPG axis involves disruption of insulin and its receptor. Therefore, to comprehensively assess the impact of 22 PAEs on various indicators of human reproductive toxicity risks, it is recommended to prioritize restricting the large-scale use of PAEs with unacceptable risks or to control their release into the natural environment through multiple recovery and treatment methods. Meanwhile, it is necessary to modify and substitute PAE molecules through approaches such as molecular modification design to reduce their impact on the three key reproductive toxicity risk endpoints identified above under PAE exposure, aiming to minimize or control the potential reproductive health risks posed by PAEs.

### 2.5. Verification and Analysis of Variability in HPG Axis Reproductive Toxicity Risks Under PAE Exposure

#### 2.5.1. Validation of Reproductive Toxicity Risks of PAEs Based on Machine Learning Methods

Using the method described in Section 3.8, the top ten most important PAEs molecular descriptors were identified (Table S6), and an XGBoost regression model for HPG axis reproductive toxicity risks under PAEs exposure was constructed. The random forest (RF) regression model used for descriptor importance selection achieved an R^2^ of 0.927. The R^2^ values of the XGBoost regression model for the training and testing sets were 0.9877 and 0.9112, respectively, with a difference of 0.0765, which is less than 0.1000. Additionally, the mean absolute error (MAE) was 1.344 and the root mean square error (RMSE) was 1.562, with MAE approximately equal to RMSE, indicating a relatively uniform error distribution. The model demonstrated good data fitting performance, reliability, and stability.

**Figure 3:**
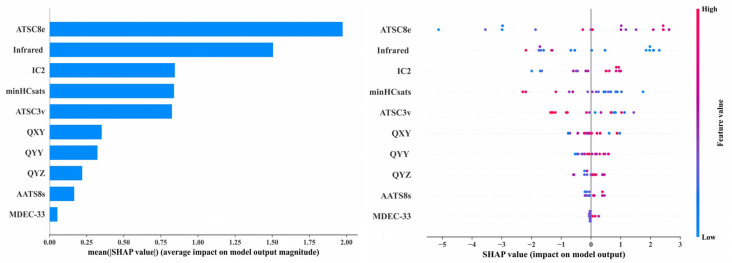
Key PAE moleculars descriptors and SHAP value plot in the XGBoost model.

Analysis of the Shapley Additive Explanations (SHAP) bar plot and swarm plot of the XGBoost regression model revealed that the descriptors Infrared (Infrared characteristic vibration spectral signal) and ATSC8e (Centered Broto–Moreau autocorrelation—lag 8/weighted by Sanderson electronegativities) had the most significant impact on the model, while the contributions of MDEC-33 (molecular distance edge between all tertiary carbons) and Q_YZ_ (the dipole moment between Y axis and Z axis) were relatively minor, and the contributions of the remaining descriptors were negligible. As shown in Figure 3, the red points corresponding to descriptor ATSC8e were primarily distributed on the right side of the x-axis, indicating that higher ATSC8e values showed a positive correlation with the first-level reproductive toxicity risk index. Conversely, the blue points corresponding to the descriptor Infrared were primarily located on the right side, indicating that lower Infrared values showed a negative correlation with the first-level reproductive toxicity risk index. Therefore, ATSC8e is positively correlated, while Infrared is negatively correlated with the first-level reproductive toxicity risk of PAEs via the HPG axis. In particular, lower ATSC8e and higher Infrared values of PAE molecules were associated with a lower first-level reproductive toxicity risk index, indicating that these corresponded to a higher reproductive toxicity risk. This study takes DUP (unacceptable risk) and DBP (acceptable risk) as examples. The ATSC8e value of DUP (−0.9188) is lower than that of DBP (−0.7283), while the Infrared value of DUP (417.3) is higher than that of DBP (362.9), indicating that the reproductive toxicity risk under DUP exposure is higher than that under DBP exposure. This finding is consistent with the results discussed in Section 3.4.1 of this manuscript. Studies have shown that the Infrared descriptor represents the characteristic vibrational Infrared spectral signals of molecules, with its values determined by molecular structure and substituent positions [43]. Therefore, the molecular structure of PAEs exerts the greatest influence on their reproductive toxicity risks, determining the intensity of the resulting toxic risks. Gu et al. [44] indicated that the molecular structure of PAEs governs their degradation rate; the slower the degradation, the longer they persist in the human body, leading to more prolonged reproductive toxicity risks. Li et al. [29] also pointed out that the number of carbon atoms and the structure of the ester side chains in PAE molecules significantly affect their reproductive toxicity risks. Hamid et al. [45] found that in vivo exposure to individual and combined PAEs significantly induced developmental lethality and malformations in zebrafish embryos. The binary mixtures of PAEs caused reproductive toxicity risks and disrupted the HPG axis pathway. The descriptor ATSC8e is positively correlated with the first-level evaluation index of PAEs’ reproductive toxicity risks, and its magnitude is determined by the molecule’s electronegativity [46], indicating that the electronegativity of PAE molecules has a significant impact on their reproductive toxicity risks. Previous studies have demonstrated that the electronegativity of PAE molecules governs hydrogen bond formation, enabling PAEs to bind to receptors via hydrogen bonds and disrupt normal HPG axis pathways [47]. The descriptor IC2 (Information content index (neighborhood symmetry of 2-order)), ranked third in importance, is positively correlated with the first-level evaluation index of PAEs’ reproductive toxicity risks. Research indicates that IC2 represents the second-order neighborhood symmetry of PAE molecules, and various symmetrical arrangements of PAEs can alter molecular structure, thereby affecting their reproductive toxicity risks [48]. Celik et al. [49] developed a QSAR model for predicting drug toxicity and found that the descriptor IC2 had a significant impact on drug toxicity. Furthermore, Karaduman et al. [50] also identified a significant correlation between IC2 and reproductive toxicity risks in their QSAR model for drug toxicity prediction. These analyses further demonstrate the reliability and accuracy of the key descriptors selected in this study.

The SHAP values of Q_YY_ (the dipole moment between Y axis and Y axis), minHCsats (Minimum atom-type H E-State: H bonded to B, Si, P, Ge, As, Se, Sn, or Pb), ATSC3v (Centered Broto–Moreau autocorrelation—lag 3/weighted by van der Waals volumes), Q_XY_ (the dipole moment between X axis and Y axis), and AATS8s (Average Broto–Moreau autocorrelation—lag 8/weighted by I-state) are all below 1. However, despite their relatively low SHAP values, ATSC3v relates to the van der Waals volume of molecules [51], while Q_YY_ and Q_XY_ pertain to molecular volume and charge distribution [52]. These descriptors still play important roles in the environmental fate, bioaccumulation, biodegradability, and toxicity of chemical pollutants [53]. MDEC-33 is closely related to the types of chemical bonds within molecules [54], and its value being well below 0.25 indicates that, compared to molecular structure, electronegativity, and charge distribution, the variety of chemical bonds in PAE molecules contributes less to their reproductive toxicity risks. In summary, the structural features, substituent positions, and electronegativity of PAE molecules significantly influence the intensity of reproductive toxicity risks mediated via the HPG axis upon exposure.

#### 2.5.2. Validation of Reproductive Toxicity Risks of PAEs Based on Charge Regularity Analysis

In this study, the charge distribution and atomic charges of 22 PAE molecules were calculated using Chem 3D version 14.0.0.17 software. The results are available at (https://github.com/Frank-XK/PAEs-charge.git) accessed on 21 April 2025. The findings indicate that the carbon atoms connecting the benzene ring on the ester side chains of PAEs carry the highest positive charges, the oxygen atoms bonded to these carbons bear the most negative charges, and all hydrogen atoms carry positive charges. The above analysis indicates that the charge distribution in PAE molecules is predominantly concentrated on their carbon–oxygen double bond structures. The electronegativity of PAE molecules is primarily determined by these carbon–oxygen double bonds, with electron transfer occurring from hydrogen atoms to carbon atoms. Based on the analysis in Section 2.5.1, it is evident that the electronegativity of PAE molecules significantly influences their reproductive toxicity risks upon exposure. Therefore, it is hypothesized that the primary factor driving PAE-induced reproductive toxicity risks via the HPG axis is their carbon–oxygen double bond structure, which facilitates effective binding between PAEs and receptors through hydrogen bonding.

Meanwhile, this study further investigates the mechanistic differences in reproductive toxicity risks of PAEs under exposure by analyzing charge distribution patterns. Three groups of PAE molecules with identical carbon numbers but differing ester side chain structures—DIPRP and DPRP, DIHP and DHP, and DIBP and DBP—were selected as examples. These molecules share the same number of carbon atoms, but their ester side chains differ, being either branched or linear. The atomic charges of these six PAE molecules were calculated to explore the relationship between the atomic charge distribution and the intensity of reproductive toxicity risks upon exposure. Based on the analysis presented in Section 2.2, when the number of carbon atoms was the same, PAE molecules with branched ester side chains exhibited greater reproductive toxicity risks than those with linear side chains. The electronegativity of PAE molecules was characterized by the average charge carried by the carbon atoms in the ester side chains (Table S7). It was found that, among PAE molecules with the same number of carbon atoms, those with branched ester side chains exhibited higher electronegativity than those with linear chains. This analysis indicates that electronegativity influences the formation of hydrogen bonds between PAEs and receptor proteins; higher electronegativity facilitates hormonal effects in the human body, thereby inducing reproductive toxicity risks [55].

## 3. Materials and Methods

### 3.1. Research Methods for Investigating Reproductive Toxicity Induced by PAE Exposure Based on the HPG Axis

#### 3.1.1. Construction of the Pathway Underlying PAEs-Induced HPG Axis Dysfunction

This study summarized previous research on PAE-induced HPG axis dysfunction [22] and constructed four hypothalamic pathways, two pituitary pathways, and two gonadal pathways.

**Hypothalamic Pathway**: Firstly, PAE molecules were found to affect the binding of E2 to estrogen receptor alpha (ERα), suppressing the secretion of kisspeptin by inducing histone H3 deacetylation at the kiss1 promoter. PAE molecules were found to disrupt the specific binding of IGF-1 to the IGF-1R/IRS-1 complex, initiating key phosphorylations within the AKT-mediated mTOR pathway to regulate the synthesis and release of kisspeptin. Subsequently, kisspeptin specifically binds to G protein-coupled receptor 54 (GPR54) on the surface of GnRH neurons, activating phospholipase C (PLC). PLC not only mediated intracellular calcium increase through the hydrolysis of inositol 1,4,5-trisphosphate (IP3) and diacylglycerol to promote GnRH secretion, but also enhanced the expression of the gene encoding GnRH by activating protein kinase C (PKC). Furthermore, PAE molecules were found to influence the release of prostaglandin E2 (PGE2) by affecting the binding of oxytocin to its receptor and the interaction between IGF-1 and the IGF-1R/IRS-1 complex. The binding of PGE2 to its receptors on GnRH neurons subsequently stimulates GnRH secretion. The hypothalamic AOP is presented in Table S8.

**Pituitary Pathway:** PAE molecules were found to affect the binding of GnRH secreted by the hypothalamus to GnRH receptors on the pituitary gland. The binding of GnRH to its receptors activated two G protein subtypes, G[q] and G[s]. G[q] and G[s] subsequently activated the PKC/MAPK and cAMP/PKA signaling pathways, respectively, leading to the activation of gonadotropin transcription factors and thereby influencing the secretion of FSH and LH. The pituitary AOP is presented in Table S8.

**Gonadal Pathway:** Previous studies have found that during the disruption of hormonal effects caused by PAEs in organisms, the estrogenic effects of PAE molecules are significantly stronger than their androgenic effects. Therefore, it is preliminarily inferred that the impact of PAE exposure on the female reproductive system may be more pronounced. Consequently, this study focuses solely on the ovary and its functional cells when investigating the reproductive toxicity risks of PAE molecules on the gonads. Firstly, the binding of INS to INSR on follicular membrane cells activates IRS-1. Activated IRS-1 subsequently triggers PI3K and MAPK signaling cascades, inducing steroid biosynthesis. However, PAE molecules can interfere with the binding of INS to INSR. Furthermore, PAE molecules can affect the binding of LH to its receptor (LHR). The LH-LHR binding couples with the G[s] protein to induce the expression of key enzymes involved in steroidogenesis via the cAMP/PKA signaling pathway. The gonadal AOP is presented in Table S8.

#### 3.1.2. Identification of Receptor Source in PAEs-Induced HPG Axis Dysfunction Pathways

To characterize the reproductive toxicity risks of PAEs, multiple reproductive toxicity pathways were constructed based on three sites: the hypothalamus, pituitary, and gonads. Twenty-two commercially available PAE molecules (Table S9) were selected as research subjects. These PAEs are frequently detected in various environmental media; they possess diverse molecular structures and are considered to exhibit a range of physiological toxicities. Among them, DEHP, DEP, DMP, DBP, BBP, and DNOP have been listed as priority pollutants by the United States Environmental Protection Agency (EPA) [56,57,58,59]. The binding energies between PAEs and receptor proteins along the HPG axis were calculated to characterize the reproductive toxicity risks of PAEs. The toxic risks and mechanisms of PAE molecules on the hypothalamus, pituitary, and gonads were investigated separately. The 3D structures of key proteins in each pathway were obtained from the UniProt protein database (https://www.uniprot.org) accessed on 21 October 2024. PAE molecules were drawn using ChemDraw 2019 software, and their 3D structures were sourced from the ChemSpider database (http://www.chemspider.com) accessed on 2 October 2024.

### 3.2. Acquisition of Dimeric Proteins in HPG Axis Reproductive Toxicity Pathways

**Figure 4:**
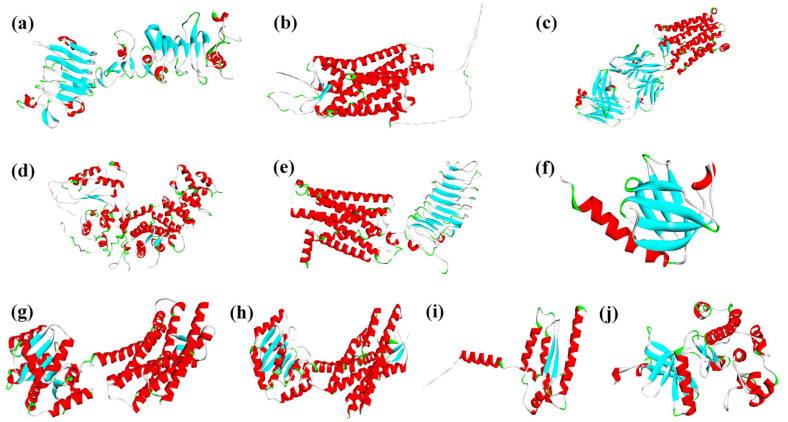
Receptor characterization of HPG axis reproductive toxicity risks under PAEs exposure (**a**): IGF-1R (ID: P08069); (**b**): GPR54 (ID: Q969F8); (**c**): PGE2R (ID: P34995); (**d**): G(q) (ID: P50148); (**e**): LHCGR (ID: P22888); (**f**): IRS-1(ID: P35568); (**g**): OxytocinR (ID: P30559); (**h**): GNRHR (ID: P30968); (**i**): G(s) (ID: O60726); (**j**): INSR (ID: P06213)). (Note: Red and blue represent alpha-helix and beta-sheet respectively).

The constructed PAEs molecular HPG axis reproductive toxicity pathways involved multiple dimeric proteins. Therefore, protein–protein docking was performed using the HDOCK server (http://hdock.phys.hust.edu.cn/) accessed on 31 October 2024 for the following pairs: IGF-1R with IRS-1, GNRHR with G[s], GNRHR with G[q], LHCGR with G[s], and INSR with IRS-1. The resulting dimeric protein structures provided initial conformations for subsequent MDs. Protein sources and identifiers are shown in Figure 4.

### 3.3. Characterization of Reproductive Toxicity Risks of PAE Molecules Based on the HPG Axis

To investigate the risks of exposure to 22 PAEs on the pathways responsible for normal reproductive functions across various parts of the HPG axis, the reproductive toxicity risks of PAEs on the hypothalamus, pituitary, and gonads were quantified. Based on the methods described in Section 3.1 and Section 3.2, binding free energies between ligand molecules and receptor proteins in each pathway were calculated using Discovery Studio 2020 software [60] and GROMACS 4.6.5 software running on a Dell PowerEdge R7425 server. These calculations were employed to characterize the reproductive toxicity risks associated with PAE exposure along the HPG axis. First, the complex was placed in a cubic box with a boundary of 0.1 nm, and SPC-type water molecules (SPC216) were added to the box along with PAE molecules. Additionally, an appropriate number of ${\mathrm{Na}}^{+}$ or ${\mathrm{Cl}}^{-}$ ions were added to maintain the overall charge neutrality of the simulation system. Subsequently, energy minimization was performed using the steepest descent method until the energy converged to 1000 kJ/mol, allowing the system to reach an equilibrium energy state. Following this, NVT temperature control and NPT pressure control simulations were conducted. Molecular dynamics equilibration simulations were performed under set temperature and pressure conditions using the leapfrog Newton integrator. Finally, binding free energies were calculated using the Molecular Mechanics/Poisson–Boltzmann Surface Area method. A larger absolute value of the binding free energy indicates stronger binding between the ligand molecule and receptor protein [61], which corresponds to a higher reproductive toxicity risk mediated by the HPG axis under PAE exposure. In this study, the absolute value of the binding free energy was used to represent the reproductive toxicity risk of PAE molecules.

### 3.4. Construction of a Graded Evaluation System for Reproductive Toxicity Indicators of the HPG Axis Under PAE Exposure

**Figure 5:**
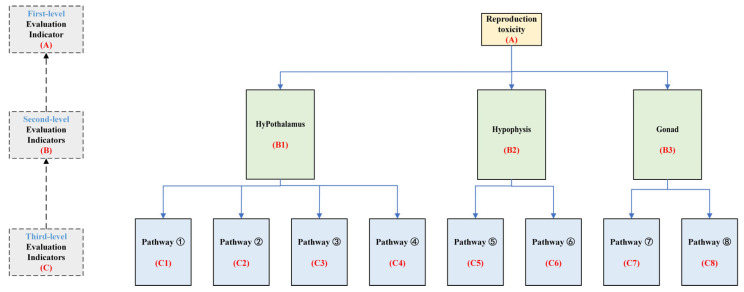
Graded evaluation system of reproductive toxicity indicators based on the HPG axis under PAE exposure.

To systematically and comprehensively analyze the reproductive toxicity risks of PAE exposure on the HPG axis, PAE molecules exhibiting significant reproductive toxicity risks, along with their key target sites and pathways, were selected. This study established a graded evaluation system for reproductive toxicity risks of the HPG axis under PAE exposure (Figure 5). The evaluation indicators are divided into three levels. The first-level indicator set A is defined as A = {B1, B2, B3}. The second-level indicator sets are defined as B1 = {C1, C2, C3, C4}, B2 = {C5, C6}, and B3 = {C7, C8}, where C represents the third-level indicators. Using subjective weighting, inverse intervalization, CRITIC weighting, and composite index methods, the adverse effects of different PAE molecules on the HPG axis and its various pathways were evaluated. This approach provides a theoretical framework and data reference for the health risk assessment of PAE molecules.

#### 3.4.1. Calculation of the Three-Level Evaluation Indicators for Reproductive Toxicity Risks of the HPG Axis Under PAE Exposure

The subjective weighting method is a qualitative approach that assigns weights to different indicators based on prior experience [62]. To comprehensively analyze the differences in reproductive toxicity risks of PAE molecules across various pathways, this study applied the subjective weighting method to assign weights to the effect values of initiating and key events in pathways ①, ②, ③, ④, ⑤, ⑥, ⑦, and ⑧, respectively. The comprehensive effect values of each pathway were then obtained to characterize the reproductive toxicity risks of PAE molecules. These comprehensive effect values serve as the third-level evaluation indicators for reproductive toxicity risks of the HPG axis under PAE exposure. According to previously published research [35], in the AOP of estrogen, androgen, and thyroid hormone effects under PAE exposure, the weight ratio between initiating events and key events is approximately 6:4. However, no relevant literature confirms the relative importance of initiating and key events in the eight pathways discussed in this study. Therefore, for analytical convenience, this study uniformly assigns weights of 5:5 to initiating and key events, which is reasonably close to the 6:4 ratio.

#### 3.4.2. Calculation of the Second-Level Evaluation Index for Reproductive Toxicity Risks of the HPG Axis Under PAE Exposure

The inverse intervalization method is a data processing technique that converts inverse indicators into positive ones and compresses data into a specified range [63]. The CRITIC weighting method objectively determines indicator weights based on the contrast intensity and conflict among evaluation indicators. It considers both the variability of indicators and their correlations, meaning that a higher numerical value does not necessarily imply greater importance. This method fully utilizes the intrinsic objective properties of the data for scientific evaluation [64].

The third-level evaluation indicators for reproductive toxicity risks of the HPG axis under PAE exposure are negative indicators. Therefore, inverse intervalization was applied to these indicators. To avoid zero values after inverse processing, which could affect subsequent machine learning model construction and analysis, the indicators were scaled to the range of 1 to 2. The specific formula is as follows:

(1) $$X_{j}^{’}=\frac{x_{max}-\;x_{j}}{x_{max}-x_{min}}+1$$

In the formula, $X_{j}^{’}$ represents the inverse intervalized value of the third-level evaluation indicator for reproductive toxicity risks of the HPG axis under exposure to the PAE molecule; $x_{j}$ is the original third-level evaluation indicator value for the PAE molecule; $x_{min}$ and $x_{max}$ denote the minimum and maximum values of the third-level evaluation indicators under PAE exposure, respectively.

Secondly, this study utilized the CRITIC weighting method within the statistical analysis platform SPSSPRO (https://www.spsspro.com/) accessed on 8 January 2025 to calculate the weights of third-level evaluation indicators for the hypothalamus, pituitary, and gonadal pathways. The third-level indicator weight sets are defined as WB1-C = {W_C1_, W_C2_, W_C3_, W_C4_}, WB2-C = {W_C5_, W_C6_}, WB3-C = {W_C7_, W_C8_}. Subsequently, the comprehensive effect values of the hypothalamus, pituitary, and gonadal sections were calculated as the second-level evaluation index for reproductive toxicity risks of the HPG axis under PAE exposure. The specific calculation steps are as follows:

(2) $$Q_{B}\;={\;W}_{C}\;\times {\;Q}_{k}$$

In the formula, $Q_{k}$ represents the specific value set of the third-level evaluation indicators, defined as $Q_{k}={\left\{X_{1},X_{2},\ldots X_{j}\right\}}^{T}$; $Q_{B}$ denotes the value set of the second-level evaluation index; and $W_{C}$ indicates the respective weights of the third-level indicators.

#### 3.4.3. Calculation of the First-Level Evaluation Index for Reproductive Toxicity Risks of the HPG Axis Under PAE Exposure

In this study, the CRITIC weighting method was employed to calculate the weights of the second-level evaluation indices for reproductive toxicity risks of the HPG axis under PAE exposure. The secondary indicator weight set is defined as W_B_ = {W_B1_, W_B2_, W_B3_}. Subsequently, the composite index method was applied to compute the comprehensive effect value of reproductive toxicity risks of the HPG axis under PAE exposure, ultimately deriving the first-level evaluation index. The specific calculation steps are as follows:

(3) $$Q_{A}\;=\;W_{B}\;\times \;Q_{m}$$

In the formula, $Q_{m}$ represents the value set of the second-level evaluation indices, defined as $Q_{m}={\left\{x_{1},x_{2}{,x}_{3}\right\}}^{T}$; $Q_{A}$ denotes the first-level evaluation index; and $W_{B}$ indicates the respective weights of the second-level evaluation index.

### 3.5. Graded Toxicity Characteristic Analysis of Reproductive Toxicity Risks on the HPG Axis Under PAE Exposure

To comprehensively compare the significant differences in reproductive toxicity risks on the HPG axis under exposure to different PAE molecules, this study conducted a systematic analysis and validated the validity of the selected significant and non-significant indicators of these effects.

FA was performed using the SPSSPRO statistical analysis platform (https://www.spsspro.com/) accessed on 18 January 2025. Dimensionality reduction was performed on the second-level evaluation index values (A-B1, A-B2, A-B3) of reproductive toxicity risks on the HPG axis induced by PAE molecules, aiming to investigate the characteristics of HPG axis reproductive toxicity risks under exposure to 22 different PAE molecules. The specific procedure was as follows: (1) the secondary indicator values of the 22 PAE molecules were treated as quantitative variables, and the 22 PAE molecules themselves were classified as categorical variables; (2) the number of principal components was set equal to the number of quantitative variables, the eigenvalue threshold was set to the default value of 1.00, and the factor rotation method was chosen as “varimax”; (3) the analysis was initiated by clicking “start,” and the factor analysis results were obtained. Since the graded evaluation indicators of reproductive toxicity risks on the HPG axis under PAE exposure are all negative indicators, a higher comprehensive score of HPG axis reproductive toxicity risks corresponds to a weaker toxic risk of PAEs on the HPG axis.

### 3.6. Analysis of Graded Indicators Characterizing Reproductive Toxicity Risks on the HPG Axis Under PAE Exposure

The RSR comprehensive evaluation method, which calculates a dimensionless statistical indicator (RSR value) from a n × m matrix through rank transformation, facilitates the comparison of differences or identification of associations [65]. This method was applied to perform dimensionality reduction on the secondary- and third-level evaluation index values of reproductive toxicity risks on the HPG axis under exposure to 22 PAEs, aiming to elucidate the characteristics and patterns of reproductive toxicity risks across different segments of the HPG axis. The analysis was conducted using the RSR comprehensive evaluation method implemented within the SPSSPRO statistical analysis platform.

### 3.7. Development of Priority Control List and Key Attention Indicator Lists for Reproductive Toxicity Risks of PAEs

To better characterize the reproductive toxicity risks on the HPG axis induced by PAE exposure and to provide a prioritized control evaluation list along with key attention indicators for PAEs’ reproductive toxicity risks, this study employed the equal interval classification method [66]. The toxic risks of 22 common environmental PAEs and their secondary- and third-level evaluation indices of HPG axis reproductive toxicity risks across various pathways were ranked from the perspectives of comprehensive toxicity risk evaluation and evaluation at different indicator levels. Furthermore, a priority control list and key focus areas for PAEs are proposed to provide theoretical guidance for managing the reproductive toxicity risks of PAEs in the environment. The operational steps of the equal interval classification method are as follows: (1) determine the maximum and minimum values of the evaluation indicators; (2) identify equal interval points a, b, c, d, and e, ensuring that these five points divide the range between the minimum and maximum values into six equal intervals, with all indicator values falling within these intervals; (3) assign scores of 6, 5, 4, 3, 2, and 1 sequentially from the smallest to the largest values based on the six equal intervals.

### 3.8. Mechanistic Analysis of Reproductive Toxicity Risks Based on the HPG Axis Under PAE Exposure

**Figure 6:**
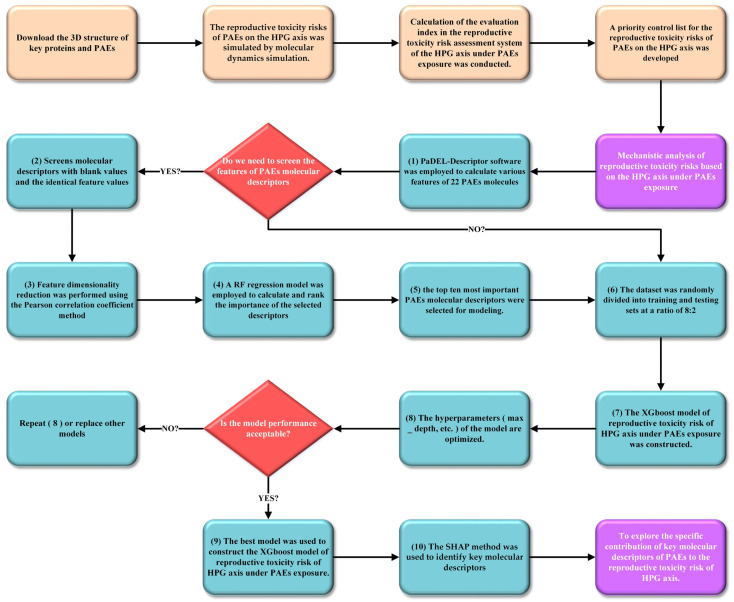
A flowchart illustrating the steps used for reproductive toxicity risks based on the HPG axis under PAE exposure constructed in this study.

To further analyze and validate the priority control list of reproductive toxicity risks based on the HPG axis under PAE exposure constructed in this study, PaDEL-Descriptor 2.21 software was employed to calculate various features of 22 PAE molecules, including geometric, electronic, physicochemical, spectral, and topological parameters. Subsequently, quantitative structure–activity relationship (QSAR) models of HPG axis reproductive toxicity risks under PAE exposure were developed using machine learning methods. To improve the training efficiency of the machine learning models, molecular descriptors of PAEs were preprocessed as follows: (1) after removing molecular descriptors with missing values and applying variance filtering to exclude descriptors with identical feature values, 946 molecular descriptors remained (946 descriptors are provided in Supplementary Materials Table S10). (2) Feature dimensionality reduction was performed using the Pearson correlation coefficient method with a threshold of 0.75. (3) A RF regression model (n_estimators = 12, random_state = 48) was employed to calculate and rank the importance of the selected descriptors, and the top ten most important PAE molecular descriptors were selected. Subsequently, the first-level evaluation index of reproductive toxicity risks based on the HPG axis under exposure to 22 PAEs, calculated as described in Section 3.4.3, was used as the dependent variable, while the ten selected PAE molecular descriptors served as independent variables. The dataset was randomly divided into training and testing sets at a ratio of 8:2 [67], comprising 17 and 5 PAE molecules in the training and testing sets, respectively. An XGBoost regression model (max_depth = 10, n_estimators = 22, subsample = 0.76) was constructed. Finally, the SHAP method was employed to identify key molecular descriptors and analyze their specific contributions to PAEs’ reproductive toxicity risks. Higher SHAP values indicated greater variable importance [68]. Detailed code is provided in Supplementary Materials Figures S1–S3. Figure 6 also provides a flowchart illustrating the steps used for reproductive toxicity risks based on the HPG axis under PAE exposure constructed in this study [69,70,71].

## 4. Conclusions

This study constructed a hierarchical evaluation system for reproductive toxicity risk indicators based on the human HPG axis under PAE exposure by coupling machine learning with the AOP approach. Through both longitudinal and cross-sectional analyses, PAE molecules with significant reproductive toxicity risks and key affected pathways were identified. Based on this hierarchical evaluation system, priority control and key toxicity indicator lists for HPG axis reproductive toxicity risks under PAE exposure were established. An XGBoost model for assessing reproductive toxicity risks of the HPG axis under PAE exposure was successfully constructed. Further analysis of PAE molecular structures revealed that (1) the intensity of reproductive toxicity risks under PAE exposure was related to the ester side chain structure and carbon number; when the carbon number was the same, PAEs with branched side chains exhibited more pronounced reproductive toxicity risks; among PAE molecules with branched side chains, a larger carbon number corresponded to weaker reproductive toxicity risks under exposure. (2) DIPRP, DMEP, DMP, DPP, and DUP were classified as PAE molecules posing unacceptable risks; particular attention should be given to the adverse effects of PAEs’ exposure on the binding processes of IGF-1 with the IGF1R/IRS-1 complex, GnRH with the GnRH/G(s) complex, and INS with INSR. (3) The PAEs descriptors most significantly associated with the intensity of reproductive toxicity risks on the HPG axis were Infrared and ATSC8e. The structure, substituent position, and electronegativity of PAE molecules significantly influenced their reproductive toxicity risks under exposure. (4) The charge distribution of PAE molecules was predominantly concentrated on their carbonyl groups, which was a key factor influencing the reproductive toxicity risks of PAEs through the HPG axis. This study established priority control and toxicity indicator attention lists for reproductive toxicity risks on the HPG axis under PAE exposure and theoretically explored the microscopic toxicity mechanisms of PAEs. These findings contribute to mitigating environmental and human health risks associated with PAE exposure, broaden the approach to investigating adverse effects of organic pollutants on organisms based on molecular properties, and provide a theoretical basis for further research on PAE-induced reproductive toxicity risks via the HPG axis. The aim is to offer new ideas and directions for targeted management of PAEs and the design of novel PAE molecular modifications. Meanwhile, the limitations of this study primarily stemmed from the inaccuracy of 3D protein structure prediction and the need to advance the physiological environment simulation. In the future, with continuous advancements in prediction and simulation technologies, further investigations into the human health risks of PAEs will be conducted.

## Figures and Tables

**Table 1:** Priority control List of reproductive toxicity risks under PAEs exposure.

Level of Risk	Scores	PAEs
Unacceptable risks	6	DIPRP
	5	DMEP, DMP, DPP, DUP
Potential risks	4	DEP, DIBP, DIHXP, DIOP
	3	BBP, DIHP, DIPP, DNOP, DNP, DTDP
Acceptable risks	2	DAP, DEHP, DHP, DPRP
	1	DBP, DIDP, DINP

**Table 2:** List of key indicators for focused attention in the assessment of PAEs reproductive toxicity risks.

Level 2			Level 3		
Index	Scores	Level of Attention	Index	Scores	Level of Attention
A-B1	1	secondary attention	A-B1-C1	5	focused attention
A-B2	4	general attention	A-B1-C2	4	general attention
A-B3	5	focused attention	A-B1-C3	3	general attention
			A-B1-C4	6	focused attention
			A-B2-C5	2	secondary attention
			A-B2-C6	4	general attention
			A-B3-C7	2	secondary attention
			A-B3-C8	6	focused attention

## Data Availability

Data are contained within the article or Supplementary Materials.

## Supplementary Materials

The following supporting information can be downloaded at: https://www.mdpi.com/article/10.3390/ijms26157389/s1.

## References

[1] Machtinger R., Gaskins A.J., Racowsky C., Mansur A., Adir M., Baccarelli A.A., Hauser R. (2018). Urinary concentrations of biomarkers of phthalates and phthalate alternatives and IVF outcomes. Environ. Int..

[2] Kashyap D., Agarwal T. (2018). Concentration and factors affecting the distribution of phthalates in the air and dust: A global scenario. Sci. Total Environ..

[3] Cao Y., Lin H., Zhang K., Xu S., Yan M., Leung K.M., Lam P.K. (2022). Microplastics: A major source of phthalate esters in aquatic envi-ronments. J. Hazard. Mater..

[4] Deng H., Li R., Yan B., Li B., Chen Q., Hu H., Shi H. (2021). PAEs and PBDEs in plastic fragments and wetland sediments in Yangtze estuary. J. Hazard. Mater..

[5] Anh H.Q., Nguyen H.M.N., Do T.Q., Tran K.Q., Minh T.B., Tran T.M. (2021). Air pollution caused by phthalates and cyclic siloxanes in Hanoi, Vietnam: Levels, distribution characteristics, and implications for inhalation exposure. Sci. Total Environ..

[6] Tran T.M., Le H.T., Minh T.B., Kannan K. (2017). Occurrence of phthalate diesters in indoor air from several Northern cities in Vietnam, and its implication for human exposure. Sci. Total Environ..

[7] Anh H.Q., Tomioka K., Tue N.M., Suzuki G., Minh T.B., Viet P.H., Takahashi S. (2019). Comprehensive analysis of 942 organic mi-cro-pollutants in settled dusts from northern Vietnam: Pollution status and implications for human exposure. J. Mater. Cycles Waste Manag..

[8] Anh H.Q., Tran T.M., Thuy N.T.T., Minh T.B., Takahashi S. (2019). Screening analysis of organic micro-pollutants in road dusts from some areas in northern Vietnam: A preliminary investigation on contamination status, potential sources, human exposure, and ecological risk. Chemosphere.

[9] Chau H.T.C., Kadokami K., Duong H.T., Kong L., Nguyen T.T., Nguyen T.Q., Ito Y. (2018). Occurrence of 1153 organic micropollutants in the aquatic environment of Vietnam. Environ. Sci. Pollut. Res..

[10] Duong H.T., Kadokami K., Pan S., Matsuura N., Nguyen T.Q. (2014). Screening and analysis of 940 organic micro-pollutants in river sediments in Vietnam using an automated identification and quantification database system for GC–MS. Chemosphere.

[11] Liu H., Huang H., Xiao X., Zhao Z., Liu C. (2021). Effects of phthalate esters (PAEs) on cell viability and Nrf2 of HepG2 and 3D-QSAR studies. Toxics.

[12] Hubinger J.C., Havery D.C. (2006). Analysis of consumer cosmetic products for phthalate esters. J. Cosmet. Sci..

[13] Bornehag C.G., Nanberg E. (2010). Phthalate exposure and asthma in children. Int. J. Androl..

[14] Gao K., Hua K., Wang S., Chen X., Zhu T. (2025). Exploring the reproductive exposure risks of phthalates and organophosphates in atmospheric particulate matter based on quantitative structure-activity relationships and network toxicology models. J. Hazard. Mater..

[15] Wang Y.X., Zeng Q., Sun Y., Yang P., Wang P., Li J., Lu W.Q. (2016). Semen phthalate metabolites, semen quality parameters and serum reproductive hormones: A cross-sectional study in China. Environ. Pollut..

[16] Long S.E., Kahn L.G., Trasande L., Jacobson M.H. (2021). Urinary phthalate metabolites and alternatives and serum sex steroid hormones among pre-and postmenopausal women from NHANES, 2013–2016. Sci. Total Environ..

[17] Mu D., Gao F., Fan Z., Shen H., Peng H., Hu J. (2015). Levels of phthalate metabolites in urine of pregnant women and risk of clinical pregnancy loss. Environ. Sci. Technol..

[18] He D., Gao S., Zhou J., Zhang L. (2024). Epidemiological Research Progress on Effects of Phthalate Exposure on Children’s Health. Adv. Clin. Med..

[19] Hashemipour M., Kelishadi R., Amin M.M., Ebrahim K. (2018). Is there any association between phthalate exposure and precocious puberty in girls? Environ. Sci. Pollut. Res..

[20] Turan S. (2021). Endocrine disrupting chemicals and bone. Best Pract. Res. Clin. Endocrinol. Metab..

[21] Lu K.-Y., Tseng F.-W., Wu C.-J., Liu P.-S. (2004). Suppression by phthalates of the calcium signaling of human nicotinic ace-tylcholine receptors in human neuroblastoma SH-SY5Y cells. Toxicology..

[22] Zhang Y., Yang Y., Tao Y., Guo X., Cui Y., Li Z. (2023). Phthalates (PAEs) and reproductive toxicity: Hypothalamic-pituitary-gonadal (HPG) axis aspects. J. Hazard. Mater..

[23] Miccoli A., Maradonna F., De Felice A., Barucchi V.C., Estonba A., Genangeli M., Carnevali O. (2017). Detection of endocrine disrupting chemicals and evidence of their effects on the HPG axis of the European anchovy Engraulis encrasicolus. Mar. Environ. Res..

[24] Zhu Q., Liu L., Zhou X., Ma M. (2019). In silico study of molecular mechanisms of action: Estrogenic disruptors among phthalate esters. Environ. Pollut..

[25] Foster P.M., Thomas L.V., Cook M.W., Walters D.G. (1983). Effect of DI-n-pentyl phthalate treatment on testicular steroidogenic enzymes and cytochrome P-450 in the rat. Toxicol. Lett..

[26] He J., Chang K., Liu S. (2021). Phthalate levels in urine of pregnant women and their associated missed abortion risk. Reprod. Biol..

[27] Clark B.J., Cochrum R.K. (2007). The steroidogenic acute regulatory protein as a target of endocrine disruption in male reproduction. Drug Metab. Rev..

[28] Pan J., Liu P., Yu X., Zhang Z., Liu J. (2024). The adverse role of endocrine disrupting chemicals in the reproductive system. Front. Endocrinol..

[29] Li X., Mo J., Zhu Q., Ni C., Wang Y., Li H., Ge R.S. (2019). The structure–activity relationship (SAR) for phthalate-mediated developmental and reproductive toxicity in males. Chemosphere.

[30] Hu X., Gu Y., Huang W., Yin D. (2016). Phthalate monoesters as markers of phthalate contamination in wild marine organisms. Environ. Pollut..

[31] Sedha S., Lee H., Singh S., Kumar S., Jain S., Ahmad A., Bajpai V.K. (2021). Reproductive toxic potential of phthalate compounds—State of art review. Pharmacol. Res..

[32] Heindel J.J., Gulati D.K., Mounce R.C., Russell S.R., Lamb J.C. (1989). Reproductive toxicity of three phthalic acid esters in a continuous breeding protocol. Fundam. Appl. Toxicol..

[33] Parkhie M.R., Webb M., Norcross M.A. (1982). Dimethoxyethyl phthalate: Embryopathy, teratogenicity, fetal metabolism and the role of zinc in the rat. Environ. Health Perspect..

[34] Health Assessment (2008). Bis (2-Methoxyethyl) Phthalate.

[35] Li Y., Yang H., He W., Li Y. (2023). Human endocrine-disrupting effects of phthalate esters through adverse outcome pathways: A comprehensive mechanism analysis. Int. J. Mol. Sci..

[36] Wu W., Zhou F., Wang Y., Ning Y., Yang J.Y., Zhou Y.K. (2017). Exposure to phthalates in children aged 5–7 years: Associations with thyroid function and insulin-like growth factors. Sci. Total Environ..

[37] Hiney J.K., Srivastava V.K., Vaden Anderson D.N., Hartzoge N.L., Dees W.L. (2018). Regulation of kisspeptin synthesis and release in the preoptic/anterior hypothalamic region of prepubertal female rats: Actions of IGF-1 and alcohol. Alcohol. Clin. Exp. Res..

[38] Hiney J.K., Srivastava V., Lara T., Dees W.L. (1997). Ethanol blocks the central action of IGF-1 to induce luteinizing hormone secretion in the prepubertal female rat. Life Sci..

[39] Shao P., Wang Y., Zhang M., Wen X., Zhang J., Xu Z., Liu T. (2019). The interference of DEHP in precocious puberty of females mediated by the hypothalamic IGF-1/PI3K/Akt/mTOR signaling pathway. Ecotoxicol. Environ. Saf..

[40] Yang R., Zheng J., Qin J., Liu S., Liu X., Gu Y., Dong R. (2023). Dibutyl phthalate affects insulin synthesis and secretion by regulating the mitochondrial apoptotic pathway and oxidative stress in rat insulinoma cells. Ecotoxicol. Environ. Saf..

[41] Hsia T.I., Huang P.C., Chen H.C., Lo Y.T.C., Chang W.T., Jou Y.Y., Huang H.B. (2022). Relationships among phthalate exposure, oxidative stress, and insulin resistance in young military soldiers: A cumulative risk assessment and mediation approach. Environ. Int..

[42] Mukherjee D., Majumder S., Moulik S.R., Pal P., Gupta S., Guha P., Kumar D. (2017). Membrane receptor cross talk in gonadotropin-, IGF-I-, and insulin-mediated steroidogenesis in fish ovary: An overview. Gen. Comp. Endocrinol..

[43] Qiu Y.L., Jiang L., Li Y. (2018). Theoretical support for the enhancement of infrared spectrum signals by derivatization of phthalic acid esters using a pharmacophore model. Spectrosc. Lett..

[44] Gu Y.Y., Wei Q., Wang L.Y., Zhang Z.M., Zhang X.Q., Sun A.L., Shi X.Z. (2021). A comprehensive study of the effects of phthalates on marine mussels: Bioconcentration, enzymatic activities and metabolomics. Mar. Pollut. Bull..

[45] Hamid N., Junaid M., Manzoor R., Jia P.P., Pei D.S. (2020). Prioritizing phthalate esters (PAEs) using experimental in vitro/vivo toxicity assays and computational in silico approaches. J. Hazard. Mater..

[46] Mali S.N., Pandey A. (2022). Balanced QSAR and molecular modeling to identify structural requirements of imidazopyridine analogues as an-ti-infective agents against trypanosomiases. J. Comput. Biophys. Chem..

[47] He W., Yang H., Pu Q., Li Y. (2022). Novel control strategies for the endocrine-disrupting effect of PAEs to pregnant women in traffic system. Sci. Total Environ..

[48] Singh P., Kumar R., Sharma B.K., Prabhakar Y.S. (2009). Topological descriptors in modeling malonyl coenzyme A decarboxylase inhibitory activity: N-Alkyl-N-(1,1,1,3,3,3-hexafluoro-2-hydroxypropylphenyl) amide derivatives. J. Enzyme Inhib. Med. Chem..

[49] Çelik F.K., Doğan S., Karaduman G. (2024). Drug-induced torsadogenicity prediction model: An explainable machine learning-driven quantita-tive structure-toxicity relationship approach. Comput. Biol. Med..

[50] Karaduman G., Kelleci Çelik F. (2023). 2D-Quantitative structure–activity relationship modeling for risk assessment of pharmacotherapy applied during pregnancy. J. Appl. Toxicol..

[51] Zhu T., Yan H., Singh R.P., Wang Y., Cheng H. (2020). QSPR study on the polyacrylate–water partition coefficients of hydrophobic organic compounds. Environ. Sci. Pollut. Res..

[52] Silverman B.D., Platt D.E. (1996). Comparative molecular moment analysis (CoMMA): 3D-QSAR without molecular superposition. J. Med. Chem..

[53] Sun Y., Das S., Brown S.R., Blevins E.R., Qu F., Ward N.A., Papish E.T. (2023). Ruthenium pincer complexes for light activated toxicity: Lipophilic groups enhance toxicity. J. Inorg. Biochem..

[54] Janairo G.I.B., Yu D.E.C., Janairo J.I.B. (2021). A machine learning regression model for the screening and design of potential SARS-CoV-2 protease inhibitors. Netw. Model. Anal. Health Inform. Bioinform..

[55] Derewenda Z.S., Lee L., Derewenda U. (1995). The occurrence of C-H-O hydrogen bonds in proteins. J. Mol. Biol..

[56] Li J., Liu B., Yu Y., Dong W. (2024). A systematic review of global distribution, sources and exposure risk of phthalate esters (PAEs) in indoor dust. J. Hazard. Mater..

[57] Liu T., Ning L., Mei C., Li S., Zheng L., Qiao P., Zhong W. (2023). Synthetic bacterial consortia enhanced the degradation of mixed priority phthalate ester pollutants. Environ. Res..

[58] Pan B., Lei J., Pan B., Tian H., Huang L. (2025). Dialogue between algorithms and soil: Machine learning unravels the mystery of phthalates pollution in soil. J. Hazard. Mater..

[59] Wang Z., Ma J., Wang T., Qin C., Hu X., Mosa A., Ling W. (2023). Environmental health risks induced by interaction between phthalic acid esters (PAEs) and biological macromolecules: A review. Chemosphere.

[60] Pawar S.S., Rohane S.H. (2021). Review on discovery studio: An important tool for molecular docking. J. Chem. Pharm. Res..

[61] Xiao B., Pu Q., Ding G., Wang Z., Li Y., Hou J. (2025). Synergistic effect of horizontal transfer of antibiotic resistance genes between bacteria exposed to microplastics and per/polyfluoroalkyl substances: An explanation from theoretical methods. J. Hazard. Mater..

[62] Xu B., Qi N., Zhou J., Li Q. (2022). Reliability assessment of highway bridges based on combined empowerment–TOPSIS method. Sustain.-Bility.

[63] Khurana U., Samulowitz H., Turaga D. (2018). Feature engineering for predictive modeling using reinforcement learning. Proceedings of the AAAI Conference on Artificial Intelligence.

[64] Krishnan A.R., Kasim M.M., Hamid R., Ghazali M.F. (2021). A modified CRITIC method to estimate the objective weights of decision criteria. Symmetry.

[65] Wang Z., Dang S., Xing Y., Li Q., Yan H. (2015). Applying rank sum ratio (RSR) to the evaluation of feeding practices behaviors, and its associations with infant health risk in Rural Lhasa, Tibet. Int. J. Environ. Res. Public Health.

[66] Jeon J.Y., Hong J.Y., Kim S.M., Lee P.J. (2015). Classification of heavy-weight floor impact sounds in multi-dwelling houses using an equal-appearing interval scale. Build. Environ..

[67] An C., Park Y.W., Ahn S.S., Han K., Kim H., Lee S.K. (2021). Radiomics machine learning study with a small sample size: Single random training-test set split may lead to unreliable results. PLoS ONE.

[68] Mosca E., Szigeti F., Tragianni S., Gallagher D., Groh G. SHAP-based explanation methods: A review for NLP interpretability. Proceedings of the 29th International Conference on Computational Linguistics.

[69] Ekpe O.D., Moon H., Pyo J., Oh J.E. (2024). Prioritization of monitoring compounds from SNTS identified organic micropollutants in con-taminated groundwater using a machine learning optimized ToxPi model. Water Res..

[70] Kang J.K., Lee D., Muambo K.E., Choi J.W., Oh J.E. (2023). Development of an embedded molecular structure-based model for prediction of micropollutant treatability in a drinking water treatment plant by machine learning from three years monitoring data. Water Res..

[71] Dávila-Santiago E., Shi C., Mahadwar G., Medeghini B., Insinga L., Hutchinson R., Good S., Jones G.D. (2022). Machine learning applications for chemical fingerprinting and environmental source tracking using non-target chemical data. Environ. Sci. Technol..

