# Supplementary material for: Reproductive Toxicity Effects of Phthalates Based on the Hypothalamic–Pituitary–Gonadal Axis: A Priority Control List Construction from Theoretical Methods
Source: Int J Mol Sci. 2025 Jul 31;26(15):7389. doi: 10.3390/ijms26157389 (PMC12347981; doi:10.3390/ijms26157389)
Supplement: Supplementary file 1 [file ijms-26-07389-s001.zip › Supplementary Materials.pdf]

Supplement Materials

Reproductive Toxicity Effects of Phthalates Based on the Hypothalamic-Pituitary-Gonadal Axis: Adverse Outcome Pathway Analysis and Priority Control List Construction

Botian Xiao [1,2,†], Hao Yang[1,2,†], Yunxiang Li [1,2], Wenwen Wang [1,2,*], Yu Li [1,2]

1 College of Environmental Science and Engineering, North China Electric Power University, Beijing, 102206, China; xbt13785387874@163.com (B.X.); yh13601614368@163.com (H.Y.); 120222232055@ncepu.edu.cn (Y.L.); liyuxx8@hotmail.com (Y.L.)

2 MOE Key Laboratory of Resources and Environmental System Optimization, North China Electric Power University, Beijing, 102206, China; e-mail@e-mail.com

* Correspondence: 53203032@ncepu.edu.cn;

† These authors contributed equally to this work.

Table S1 Calculation table of hierarchical indicators for PAEs-induced reproductive toxicity effects on the HPG axis

Level of Evaluation	Level 3								Level 2			Level 1
Indicators of Evaluation / PAEs	Passway ①	Passway ②	Passway ③	Passway ④	Passway ⑤	Passway ⑥	Passway ⑦	Passway ⑧	Hypothalamic	Pituitary	Gonadal	Reproductive Toxicity
Abbreviations of Indicator	A-B1-C1	A-B1-C2	A-B1-C3	A-B1-C4	A-B2-C5	A-B2-C6	A-B3-C7	A-B3-C8	A-B1	A-B2	A-B3	A
Weights	20.90%	27.30%	30.90%	20.90%	47.01%	52.99%	55.90%	44.10%	30.36%	30.21%	1%	–
BBP	79.312	97.059	124.496	72.850	105.921	44.923	90.902	28.670	96.766	73.598	63.455	76.634
DAP	90.852	104.822	120.802	84.390	118.214	78.611	86.265	5.975	102.568	97.228	50.854	80.567
DBP	97.940	105.335	121.962	91.478	102.162	43.392	110.683	22.496	106.029	71.020	71.790	81.954
DEHP	91.267	91.297	125.368	84.805	110.576	93.986	54.561	31.096	100.459	101.785	44.212	78.685
DEP	86.690	101.204	129.647	80.228	80.738	70.336	63.570	22.888	102.573	75.226	45.628	71.861
DHP	86.663	94.683	136.237	80.201	119.135	44.472	93.708	21.381	102.817	79.571	61.809	79.627
DIBP	87.310	88.420	136.928	80.848	105.544	59.368	57.137	24.699	101.591	81.076	42.830	72.227
DIDP	91.829	84.058	125.900	85.367	120.557	72.738	91.723	30.180	98.882	95.218	64.580	84.252
DIHP	81.583	95.741	110.831	75.121	90.685	70.905	77.161	25.748	93.133	80.204	54.486	73.991
DIHXP	84.024	96.128	124.735	77.562	98.586	62.087	62.043	16.264	98.555	79.245	41.853	70.367
DINP	90.186	99.901	142.314	83.724	135.628	45.248	89.303	17.956	107.592	87.736	57.837	81.978
DIOP	93.225	100.605	117.673	86.763	86.237	49.599	72.225	20.419	101.442	66.822	49.377	70.456
DIPP	100.188	96.065	107.904	93.726	90.076	30.575	100.868	21.164	100.094	58.547	65.716	73.989
DIPRP	86.526	92.855	123.004	80.064	79.991	2.853	78.023	24.053	98.172	39.116	54.220	63.003
DMEP	87.154	105.915	111.618	80.692	92.616	48.149	64.711	23.218	98.483	69.053	46.411	69.063
DMP	85.627	99.372	113.508	79.165	84.029	30.743	85.207	20.952	96.642	55.793	56.868	68.620
DNOP	99.542	108.107	118.851	93.080	69.716	83.919	70.855	26.129	106.495	77.242	51.129	75.830
DNP	83.028	104.645	153.817	76.566	93.175	49.296	86.385	9.463	109.449	69.924	52.460	75.040
DPP	96.147	111.873	122.494	89.685	81.396	37.155	57.848	22.936	107.229	57.953	42.450	66.803
DPRP	83.248	93.392	123.969	76.786	98.093	43.230	106.580	27.793	97.247	69.021	71.833	78.700
DTDP	76.409	107.516	114.194	69.947	98.957	53.902	77.873	35.555	95.225	75.082	59.209	74.941
DUP	95.641	96.374	127.508	89.179	45.471	29.781	95.025	30.383	104.335	37.157	66.515	69.129

Table S2 Comprehensive FA scores and rankings of secondary indicators for HPG axis reproductive toxicity effects under PAEs exposure

Ranking	FA Composite Score	PAEs	Ranking	FA Composite Score	PAEs
1	1.059	DBP	12	-0.098	DUP
2	0.992	DINP	13	-0.230	DEP
3	0.737	DIDP	14	-0.264	DTDP
4	0.575	DHP	15	-0.291	DIBP
5	0.508	DAP	16	-0.362	DIOP
6	0.499	DNP	17	-0.387	DPP
7	0.372	DNOP	18	-0.515	DIHP
8	0.292	DPRP	19	-0.625	DIHXP
9	0.161	DEHP	20	-0.678	DMEP
10	0.022	DIPP	21	-0.700	DMP
11	0.007	BBP	22	-1.078	DIPRP

Table S3 LogKow values of the four PAEs molecules with the strongest and weakest reproductive toxicity effects

PAEs	logKow	PAEs	logKow
DIDP	8.50	DIPRP	1.63
DINP	7.52	DPP	3.74
DBP	2.76	DMP	-0.19
DAP	1.77	DEMP	-0.74

Table S4 Average values of second and third-level evaluation indicators in the PAEs HPG axis reproductive toxicity effect assessment system

Passway	Average value	Passway	Average value
A-B1-C1	88.836	A-B3-C7	80.575
A-B1-C2	98.880	A-B3-C8	23.155
A-B1-C3	124.261	A-B1	101.172
A-B1-C4	82.374	A-B2	72.619
A-B2-C5	95.796	A-B3	55.251
A-B2-C6	52.058		

Table S5 RSR fitting rankings of second and third-level evaluation indicators for reproductive toxicity effects under PAEs exposure

Evaluation level	Passway	RSR ranking	RSR score	Classification level
Level 2	A-B3	1	0.988	III
	A-B2	2	0.653	II
	A-B1	3	0.350	II
Level 3	A-B1-C4	1	1.076	III
	A-B1-C1	2	0.793	II
	A-B1-C2	3	0.571	II
	A-B1-C3	4	0.349	II

A-B2-C6	1	0.977	III
A-B2-C5	2	0.523	II
A-B3-C8	1	1.000	III
A-B3-C7	2	0.500	II

Table S6 Ten PAEs molecular descriptors selected based on pearson correlation coefficient method and random forest regression model

Name of the molecular descriptor	Abbreviation
The dipole moment between Y axis and Y axis	Q_{YY}
The dipole moment between X axis and Y axis	Q_{XY}
The dipole moment between Y axis and Z axis	Q_{YZ}
Infrared characteristic vibration spectral signal	Infrared
Average Broto-Moreau autocorrelation - lag 8 / weighted by I-state	AATS8s
Centered Broto-Moreau autocorrelation - lag 3 / weighted by van der Waals volumes	ATSC3v
Centered Broto-Moreau autocorrelation - lag 8 / weighted by Sanderson electronegativities	ATSC8e
Minimum atom-type H E-State: H bonded to B, Si, P, Ge, As, Se, Sn or Pb	minHCsats
Information content index (neighborhood symmetry of 2-order)	IC2
Molecular distance edge between all tertiary carbons	MDEC-33

Table S7 Average charge number of carbon atoms in the ester side chains of PAEs Molecules

PAEs	Average charge number
DIPRP	-0.0233
DPRP	-0.0168
DIHP	-0.0458
DHP	-0.0410
DIBP	-0.0294
DBP	-0.0288

Table S8 Pathways of PAEs molecules inducing dysfunction of the HPG axis

Parts	Pathway	Initiating event	Key event
Hypothalamic	Pathway①	IGF-1 and IGF-1-IRS-1	kisspeptin and GPR54
	Pathway②	E2 and ERα	kisspeptin and GPR54
	Pathway③	Oxytocin and OxytocinR	PGE2 and PGE2R
	Pathway④	IGF-1 and IGF-1-IRS-1	PGE2 and PGE2R
Pituitary	Pathway⑤	GNRH and GNRHR	GNRH and GNRHR-G(q)
	Pathway⑥	GNRH and GNRHR	GNRH and GNRHR-G(s)
Gonadal	Pathway⑦	LH and LHCGR	LH and LHCGR-G(s)
	Pathway⑧	INS and INSR	INS and INSR-IRS-1

Table S9 Names and information of 22 PEAs

PAEs	Name	Molecular formula	CAS
BBP	Benzyl butyl phthalate	C19H20O4	85-68-7
DAP	Diallyl phthalate	C14H14O4	131-17-9
DBP	Dibutyl Phthalate	C16H22O4	84-74-2
DEHP	Di(2-ethylhexyl) phthalate	C24H38O4	117-81-7
DEP	Diethyl Phthalate	C12H14O4	84-66-2
DHP	Dihexyl phthalate	C20H30O4	84-75-3
DIBP	Diisobutyl phthalate	C16H22O4	84-69-5
DIDP	Diisodecyl phthalate	C28H46O4	26761-40-0
DIHP	Dihexyl phthalate	C20H30O4	68515-50-4
DIHXP	Diisohexyl phthalate	C20H30O4	71850-09-4
DINP	Diisononyl phthalate	C26H42O4	28553-12-0
DIOP	Diisooctyl phthalate	C24H38O4	27554-26-3
DIPP	Diisopentyl phthalate	C18H26O4	605-50-5
DIPRP	Diisopropyl phthalate	C14H18O4	605-45-8
DMEP	Bis(2-methoxyethyl) phthalate	C14H18O6	117-82-8
DMP	Dimethyl Phthalate	C10H10O4	131-11-3
DNOP	Dioctyl phthalate	C24H38O4	117-84-0
DNP	Dinonyl phthalate	C26H42O4	84-76-4
DPP	Dipentyl phthalate	C18H26O4	131-18-0
DPRP	Di-n-propylphthalate	C14H18O4	131-16-8
DTDP	Ditridecyl phthalate	C34H58O4	119-06-2
DUP	Diundecyl phthalate	C30H50O4	3648-20-2

```python
import pandas as pd
import numpy as np
from scipy.stats import pearsonr
import pandas as pd
import numpy as np
from sklearn.feature_selection import VarianceThreshold
import pandas as pd
from sklearn.datasets import load_breast_cancer
from sklearn.feature_selection import VarianceThreshold

data = pd.read_excel("C://Users//WIN11//Desktop//新文章//msf.xlsx")
X = data.iloc[:,0:]

selector = VarianceThreshold(threshold=0.01)
X_new = selector.fit_transform(X)

print("原始特征数：", X.shape[1])
print("过滤后的特征数：", X_new.shape[1])
print("选择的特征：", X.columns[selector.get_support()])
```

```python
df1 = pd.DataFrame(X.columns[selector.get_support()])
writer = pd.ExcelWriter('C://Users//WIN11//Desktop//新文章//aaa1.xlsx')
df1.to_excel(writer,'page_1',float_format='%.5f')
writer.save()
```

```python
dataSet = pd.read_excel("C://Users//WIN11//Desktop//新文章//FangC.xlsx")
pearson_result=dataSet.corr()
pearson_result.to_excel("C://Users//WIN11//Desktop//新文章//pearson.xlsx")
```

```python
def excuteFilter(x,pearson_result,dataSet,path):
    header=pearson_result.columns
    data = pearson_result.values
    indices = np.triu_indices_from(data)
    [rows, cols] = data.shape
    addlist = []
    removelist = []
    removeindex = []
    for i in range(rows):
        if header[i] not in removelist:
            for j in range(cols):
                if j < i:
                    if data[i,j]>x or data[i,j]<-x:
                        if header[j] in addlist:
                            if header[i] not in removelist:
                                removelist.append(header[i])
                            continue
                        if header[i] not in addlist:
                            addlist.append(header[i])
                        if header[j] not in removelist:
                            removelist.append(header[j])
    retainList = []
    for col in header:
        if col not in removelist:
            retainList.append(col)
            print(col)
    dataSet[retainList].to_excel(path,index = False)
```

```python
excuteFilter(0.75,pearson_result,dataSet,"C://Users//WIN11//Desktop//新文章//retainDataSet.xlsx")
```

Figure S1 Code for feature descriptor selection

```
from sklearn.ensemble import RandomForestRegressor
from sklearn.model_selection import train_test_split
from sklearn.metrics import mean_squared_error
import pandas as pd
import numpy as np
from sklearn.metrics import r2_score
import matplotlib.pyplot as plt
import warnings
warnings.filterwarnings("ignore")
import os

data = pd.read_excel("C://Users//WIN11//Desktop//新文章//RF.xlsx")

X = data.iloc[:, 1:-1]  # Features (from column B to BCO, rows 2 to 19)
y = data.iloc[:, -1]    # Target (column BCP, rows 2 to 1+9)

for i in range(1,100,1):
    model = RandomForestRegressor(n_estimators=12, random_state=48)
    model.fit(X, y)
    y_pred = model.predict(X)
    R2 = r2_score(y, y_pred)
    print(R2)
```

```
model = RandomForestRegressor(n_estimators=12, random_state=48)
model.fit(X, y)
```

```
y_pred = model.predict(X)
y_pred
```

```
from sklearn.metrics import r2_score
R2 = r2_score(y, y_pred)
R2
```

```
feature_importances = model.feature_importances_
print(feature_importances)
```

```
from sklearn.feature_selection import RFE
#from sklearn.model_selection import cross_val_score
score = []
for i in range(1, 18, 1):
    X_wrapper = RFE(model, n_features_to_select=i, step=1).fit_transform(X, y)
    model.fit(X_wrapper, y)
    y_pred1 = model.predict(X_wrapper)
    R2 = r2_score(y, y_pred1)
    score.append(R2)
print(max(score ), (score .index(max(score ))*1)+1)
print(score )
plt.figure(figsize=[20, 5])
plt.plot(range(1, 18, 1), score )
plt.xticks(range(1, 18, 1))

plt.show()
```

Figure S2 Code for calculating feature importance using Random Forest

```python
import numpy as np
import pandas as pd
import matplotlib.pyplot as plt
pd.set_option('display.max_rows',None)
pd.set_option('display.max_columns',None)

data=pd.read_excel('C:\\Users\\HP\\Desktop\\PAEs\\新文章\\新文章\\T1-python.xlsx')

X=data.iloc[:,2:]
Y=data.iloc[:,1]

from sklearn.model_selection import train_test_split
x_train,x_test,y_train,y_test=train_test_split(X,Y,test_size = 0.2,random_state=16)

from xgboost import XGBRegressor
import xgboost as xgb
from sklearn.metrics import mean_squared_error
from sklearn.metrics import r2_score
```

```python
for i in range(1,100):
    xgb_model=xgb.XGBRegressor(
        random_state=0,
        max_depth=10,
        n_estimators=22,
        subsample=0.76
    )
    xgb_model.fit(x_train,y_train)
    y_pred = xgb_model.predict(x_test)

    score = r2_score(y_test,y_pred)


    print("i=" +str(i)+"得分",score)
```

```python
xgb_model=xgb.XGBRegressor(
    random_state=0,
        max_depth=10,
        n_estimators=22,
        subsample=0.76
    )
xgb_model.fit(x_train,y_train)
y_pred=xgb_model.predict(x_test)
R2 = r2_score(y_test,y_pred)
```

```python
MSE = mean_squared_error(y_test,y_pred)
```

Figure S3 XGBoost regression model for reproductive toxicity effects of PAEs
